# Effects of fermented soybean meal substitution for fish meal on intestinal flora and intestinal health in pearl gentian grouper

**DOI:** 10.3389/fphys.2023.1194071

**Published:** 2023-07-03

**Authors:** Aobo Pang, Cong Peng, Ruitao Xie, Zhuoduo Wang, Beiping Tan, Tingting Wang, Wei Zhang

**Affiliations:** ^1^ Laboratory of Aquatic Animal Nutrition and Feed, College of Fisheries, Guangdong Ocean University, Zhanjiang, Guangdong, China; ^2^ Aquatic Animals Precision Nutrition and High Efficiency Feed Engineering Research Center of Guangdong Province, Zhanjiang, Guangdong, China; ^3^ Key Laboratory of Aquatic, Livestock and Poultry Feed Science and Technology in South China, Ministry of Agriculture, Zhanjiang, Guangdong, China; ^4^ Guangdong Evergreen Feed Industry Co, Ltd., Zhangjiang, China

**Keywords:** fermented soybean meal, growth performance, intestinal morphology, intestinal inflammation, intestinal flora, Epinephelus fuscoguttatus♀ × E. lanceolatus♂

## Abstract

This study explored the role of replacing fish meal protein with fermented soybean meal (FSBM) protein on the growth performance and intestinal morphology, immunity, and microbiota of the pearl gentian grouper (*Epinephelus fuscoguttatus♀ × E. lanceolatus♂*). Three isonitrogenous and isolipidic diets with increasing levels of FSBM (0%, 20% and 40%; referred to as FM, FSBM20 and FSBM40 diets, respectively) as a replacement for fish meal were selected for this study. The pearl gentian grouper were fed these diets for 10 weeks. The findings revealed that the growth of fish fed the FSBM diets (FSBM20 and FSBM40) were remarkably lower than the fish fed the FM diet. Pathological manifestations of intestinal inflammation, such as shortened intestinal mucosal folds and thickened lamina propria, were observed in the fish fed the FSBM diets. Moreover, the gene expression levels of *IL1β*, *IL12*, *IL17*, and *TNFα* were remarkably upregulated in fish fed the FSBM40 diet, in contrast to the gene expression levels of *IL4, IL5, IL10,* and *TGFβ1*, which were remarkably downregulated (*p* < 0.05). The FSBM diets significantly affected the stability of the fish gut microbiota. *Photobacterium* was the dominant phylum in all experimental groups, and the proportion of these bacteria gradually decreased with increasing FSBM substitution. The composition of intestinal flora at the genus level was not the same in the three experimental groups, with a richer composition of intestinal bacteria detected in the FSBM20 and FSBM40 groups (*p* < 0.05). The correlation between intestinal flora balance and immune gene expression revealed that only *Photobacterium* was negatively correlated with the above upregulated genes, while other bacteria were positively correlated with these pro-inflammatory factors (*p* < 0.05). *Photobacterium* was positively correlated with the above downregulated genes, while other bacteria were negatively correlated with these anti-inflammatory factors (*p* < 0.05). In conclusion, high levels of substitution of FSBM for fish meal causes intestinal inflammation in pearl gentian grouper. This is likely associated with changes to the intestinal flora. More attention should be paid to the negative role of dietary FSBM on intestinal flora.

## 1 Introduction

As the scale of aquaculture continues to expand, the feed industry’s demand for raw protein materials such as fish meal continues to increase. Because fish meal is expensive and difficult to store, it is often substituted with other raw materials in the feed production process to save costs. Among the various plant protein sources, soybean meal is inexpensive and has a high crude protein content; however, it has various anti-nutritional factors (ANFs), thus limiting its application in feeding (Shang et al., 2016). After microbial fermentation, the soybean protein in soybean meal is effectively degraded into small peptide proteins and probiotics, oligo peptides, lactic acid and other active substances are produced. The resulting fermented soybean meal (FSBM) has significantly fewer ANFs, better palatability and better digestion and absorption (Hubert et al., 2008).

Replacing fish meal with FSBM in aquafeeds has been reported to have different influences on various fish. Rahimnejad et al. (2021) found that the growth performance of *Lateolabrax maculatus* reduced with increasing levels of FSBM in the diet, and the substitution of FSBM for fish meal in the diet triggered intestinal inflammation. However, the substitution of FSBM for 15%–75% of fish meal in the diet was found to have no significant effect on the intestinal tissues of *Larimichthys crocea* (Wang et al., 2019). A related study of *Epinephelus coioides* found that replacing 40% of dietary fish meal with FSBM inhibited fish growth on days 14, 28, 42, 56, 70 and 84, but had no significant effect when the replacement level was ≤20% (Shiu et al., 2015). The existing studies suggest that investigation of the effects of intestinal microbes and their metabolites on the host, in terms of nutrient metabolism, digestive enzyme activity and intestinal immunity, is extremely valuable (Jia et al., 2021).

The intestinal flora forms a complex micro-ecosystem important to the health of the host. While the host can act as a manipulator of this ecosystem, it is difficult to control each species individually (Coyte et al., 2015). The formation of the intestinal flora of fish is mainly determined by the aquatic system and the ingested bait. There are basically no bacteria in fish eggs during hatching, and after hatching, the larvae are exposed to water and bait; through interaction with the intestinal mucosa of the larvae, some bacteria that can adapt to the intestinal environment are colonized in the intestinal epithelium and gradually form the intestinal flora (Pérez et al., 2010). The intestines are an immune organ and a key organ for nutrient digestion and absorption; intestinal health is influenced by the intestinal flora, and intestinal health affects the host’s bodily functions and health. The intestinal flora of fish is unique, and the intestinal flora determines the structure and function of the intestinal microecological system. The intestinal flora is not only involved in nutrient digestion and absorption but is responsible for the body’s immune defence function (Kononova et al., 2019). As a stable ecological community, the dominant flora will exclude foreign bacteria and form a biological barrier to promote the immunity of the fish intestine. Flora species bind to the epithelial cells of the intestinal mucosa and develop different mechanisms to adapt to the intestinal environment and defend against foreign bacteria. Disordered structure of the intestinal flora is directly implicated in intestinal diseases such as intestinal inflammation (Sanmukh et al., 2012).

The pearl gentian grouper (*Epinephelus fuscoguttatus♀ × E. lanceolatus♂*) is an important species for artificial culture in China, which with high nutritional value, good sales prospects, and strong resistance to diseases; it is an important species for artificial culture in China (Wang et al., 2022). There is a lack of specific studies on the effect of FSBM in diets on intestinal health and intestinal flora of pearl gentian grouper. This study showed the role of two levels of FSBM diets on growth performance and intestinal morphology, immunity, and microbiota of this fish, and performed correlation analysis of intestinal flora changes and inflammatory cytokine expression, which will help to further explore the causes and mechanisms of soy protein-induced enteritis.

## 2 Materials and methods

### 2.1 Experimental diets

The control diet (FM) consisted of 50% fish meal, while 20% (FSBM20) and 40% (FSBM40) of the fish meal were replaced with FSBM in the other two experimental diets, respectively. The crude protein and ether extract contents of these three diets were equal at 50% and 10%, respectively, and equal lysine and methionine levels were maintained in each diet by amino acid addition. The FSBM was prepared by the fermentation of *Bacillus subtilis* and purchased from Foshan CJ Biotechnology Co. Ltd. (Foshan, China). Table 1 shows the diet formulation and approximate compositions. Some of the ingredients of the formulation were pulverized with a grinder until they could pass through a sieve with an aperture of 0.25 mm. The ingredients were mixed well according to the recipe and then the appropriate amount of water was added and stirred to mix. The mixture was obtained by passing through a granulator with diameters of 2.0 mm and 3.0 mm. It was air dried to remove 90% of the water and stored in a refrigerator at a temperature of −20°C.

**TABLE 1:** Formulation and proximate composition of the experimental diets (%, dry matter).

Ingredients (%)	Diets		
	FM	FSBM20	FSBM40
Fish meal	50.00	40.00	30.00
Fermented soybean meal	0.00	11.94	0.00
Vital wheat gluten	5.00	5.00	5.00
Wheat flour	18.00	18.00	18.00
Casein	4.60	4.60	4.60
Gelatin	1.00	1.00	1.00
Fish oil	3.02	3.76	4.49
Soybean oil	2.00	2.00	2.00
Soybean lecithin	2.00	2.00	2.00
Microcrystalline cellulose	11.48	8.67	5.84
Calcium monophosphate	1.50	1.50	1.50
Ascorbic acid	0.05	0.05	0.05
Choline chloride	0.50	0.50	0.50
Vitamin premix^a^	0.30	0.30	0.30
Mineral premix^b^	0.50	0.50	0.50
Ethoxyquin	0.05	0.05	0.05
Lysine^c^	0.00	0.06	0.13
Methionine^c^	0.00	0.07	0.15
Proximate composition) (%, dry matter)			
Crude protein	50.97	50.82	50.45
Crude lipid	10.15	10.38	10.54

^a^ Vitamin premix consisted of (g/kg premix): VB_1_ 17.00 g, VB_2_ 16.67 g, VB_6_ 33.33 g, VB_12_ 0.07 g, VK, 3.33 g, VE, 66.00 g, retinyl acetate 6.67 g, VD, 33.33 g, nicotinic acid 67.33 g, D-calcium pantothenate 40.67 g, biotin 16.67 g, folic acid 4.17 g, inositol 102.04 g, cellulose 592.72 g.

^b^ Mineral premix consisted of (g/kg premix): FeSO_4_·H_2_O 18.785 g ZnSO_4_·H_2_O 32.0991 g, MgSO_4_·H_2_O 65.1927 g, CuSO_5_·5H_2_O 11.0721 g, CoCl_2_·6H_2_O (10%) 5.5555 g, KIO_3_ 0.0213 g, KCl, 22.7411 g, Na_2_SeO_3_ (10%) 0.5555 g, zeolite powder 843.9777 g.

^c^ Lysine and Methionine were added to balance amino acid with FM, control group.

### 2.2 Feeding management

Juvenile pearl gentian grouper from Zhanjiang, Guangdong, China were raised for 1 week under experimental conditions and fed a commercial diet with a crude protein content ≥520 g/kg and crude ether extract content ≥130 g/kg. All 720 fish (12.55 ± 0.06 g) were casually divided into 12 tanks each with a capacity of 1000 L of water (4 replicates per group); there were 60 fish per tank. During the 10-week experiment, feed was given daily at 8:00 and 16:00 until the fish were satiated. The experimental fish were kept in culture tanks provided by Guangdong Ocean University. The water was changed every day. All tanks were supplied with airstones to ensure dissolved oxygen in the water column was not <7 mg L^−1^. The water was kept at 29°C ± 1°C.

### 2.3 Sample collection

After 10 weeks, all fish were weighed and their weight gain rate (WGR), specific growth rate (SGR), feed conversion ratio (FCR), and survival rate (SR) were calculated after starvation for 24 h. Subsequently, eugenol (1:10,000) was added to each tank to anesthetize the fish before sampling. 12 randomly selected fish per tank were dissected; 1 cm long samples of the distal intestines were taken from three of these fish and the intestines were soaked in 4% formaldehyde solution for histology analysis. Some of the intestinal tissues were packed in tubes with RNA latter, stored at 4°C overnight, then stored at −80°C prior to RNA extraction. To analyse the intestinal microbiota, the intestines from three fish per tank were stored at −80°C prior to 16S rDNA sequencing detection.

### 2.4 Intestinal pathology

The intestines fixed with formaldehyde were dehydrated with ethanol and embedded in paraffin. Sections were then obtained with a microtome and were stained with haematoxylin-eosin. Stained sections were observed using a microscope (Olympus CKX41, Tokyo, Japan). Observations included fold height and width, lamina propria width, and microvilli length.

### 2.5 RNA extraction and qRT-PCR

RNA was extracted from the distal intestinal tissue using Trizol reagent according to the instructions. Qualified RNA was checked using NanoDrop 2000 (Thermo Fisher Scientific, America) and electrophoresis. Qualified RNA is reverse transcribed to cDNA according to kit requirements (Evo M-MLV, Takara, Japan). Primer sequences were referenced to the PacBio SMART Pearl gentian distal intestinal tissue full-length transcriptome sequencing database from our laboratory. β-actin was used as an internal reference gene. Primer parameters are shown in Table 2. Real-time quantitative PCR was performed on a PCR Mastercycler (ep realplex, Germany). Relative expression levels of genes were determined by the 2^−△△Ct^ method (Livak and Schmittgen, 2001).

**TABLE 2:** PCR primers for mRNA expression of intestinal immune-related genes in grouper.

Gene	Forward 5′-3′	Revise 3′-5′	Size (bp)
*IL1β*	AAG​GTG​GAC​GCC​AAC​AGA​CA	GTT​CAC​TGC​AGG​CTC​AGG​GA	153
*IL12*	GAC​GGA​GCA​TTT​CCT​GGT​GG	TGC​TCC​AAG​AGC​TCG​GGT​AA	172
*IL17*	GAG​AGG​ACG​GTG​TCT​GTG​TGG	CAT​GCA​CAG​TTG​AGG​GTG​TGG	101
*TNFα*	AAC​TGT​GTG​TCC​CCA​CTG​CC	CCA​CAG​ATG​GCC​CAG​GTC​AT	81
*IL4*	GCA​GTG​AGT​GAA​GCC​ATC​GC	TGC​AGT​TCC​TGA​TAG​CGC​GA	146
*IL5*	GGC​CAA​CAG​TCA​AGA​TGT​CTG​CC	GAA​TGA​CCA​GGA​GCA​GTT​CAG​TGT	160
*IL10*	ACA​CAG​CGC​TGC​TAG​ACG​AG	TAG​ACT​TGT​GCC​ACG​ACG​GG	142
*TGFβ1*	CTT​CTC​CTC​CTC​CTC​GCT​GC	GAT​GTT​GCT​GAG​GGC​TTC​GC	195
*β-actin*	GGC​TAC​TCC​TTC​ACC​ACC​ACA	TCT​CCA​AGG​CAA​CGG​GTC​T	188

Note: *IL*, interleukin; *TNF*, tumor necrosis factor; *TGF*, transforming growth factor.

### 2.6 16S rDNA sequencing

Total DNA was extracted from the distal intestine using the E. Z.N.A.TM kit. The extracted DNA was checked for eligibility by electrophoresis. The primers used to amplify the above DNA were 338F: ACT​CCT​ACG​GGA​GGC​AGC​A and 806R: GGACTACHVGGGTATCA. PCR reaction parameters were 98°C, 1 min; 98°C, 10 s; 50°C, 30 s; 72°C, 30 s; 30 cycles, 72°C, 5 min. High-throughput sequencing was performed on the Illumina platform. Cutadapt was used to process the high-throughput sequencing data, and then Uparse was used to cluster sequences with similarities ≥97% in each sample to the corresponding operational taxonomic units (OTUs). Finally, annotation analysis was performed using the software.

### 2.7 Statistical analysis

The data were expressed as the mean and standard deviation. When the one-way analysis of variance (ANOVA) was remarkably significant (*p* < 0.05), the Turkey multiple comparison test was conducted to define the significant differences between the groups. The WGR, SGR, FCR, SR were calculated by the following formulas:

Weight gain (WG, %) = 100 × (final weight - initial weight)/initial weight; Specific growth rate (SGR, %) = 100 × [ln (finial weight) - ln (initial weight)]/days; Feed conversion ratio (FCR) = feed intake/(final weight - initial weight); Survival rate (SR, %) = 100 × (final fish number of fish/initial fish number of fish)

## 3 Results

### 3.1 Growth performance

**FIGURE 1:**
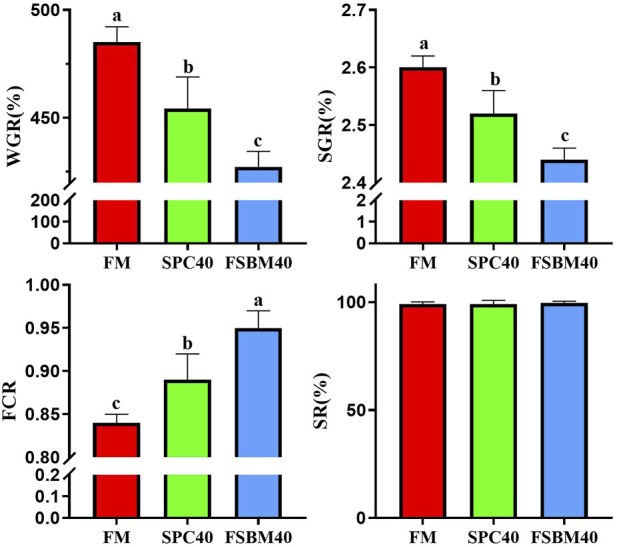
Growth performance of pearl gentian grouper fed different levels of FSBM diets (n = 4). Note: Marked with the same letter inicates no significant difference between groups.

As the content of FSBM in the diet increased, the WGR and SGR of fish in the FSBM20 and FSBM40 groups decreased remarkably and progressively compared to the FM group (*p* < 0.05). Accordingly, the FCR increased progressively (*p* < 0.05). However, differing FSBM contents did not affect the SR of fish in the experiment (Figure 1).

### 3.2 Intestinal histology

**FIGURE 2:**
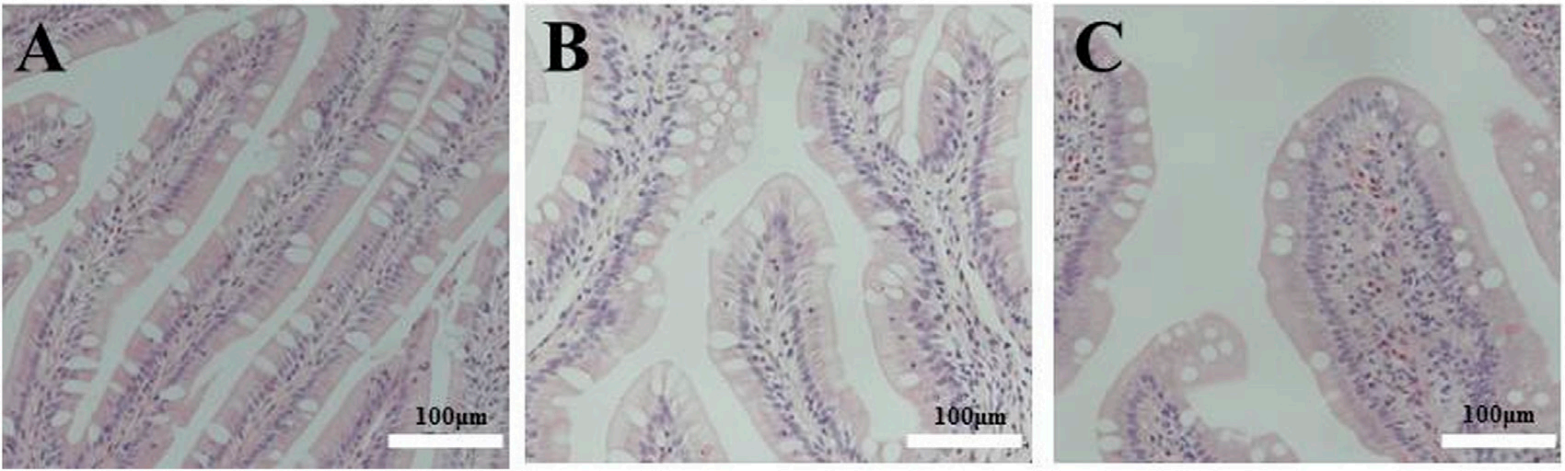
Pathological sections of the distal intestine of the pearl gentian grouper. Note: A, FM; B, FSBM20; C, FSBM40.

As shown in Figure 2, longer and wider folds and thicker lamina propria were observed in the tissue sections of the distal intestine of fish fed FSBM diets. This indicates that the FSBM20 and FSBM40 diets triggered intestinal inflammation and the pathological phenotype of intestinal inflammation became more pronounced with increasing FSBM substitution levels.

### 3.3 Inflammatory genes expression

The findings for inflammatory cytokine gene expression are shown in Table 3 and Table 4. Among the pro-inflammatory genes, no changes in *IL*12, *IL*17, and *TNFα* expression were observed in the intestines of fish in the FM group and FSBM20 group (*p* > 0.05). However, *IL1β* was remarkably upregulated in the FSBM20 group (*p* < 0.05). The above four genes were all remarkably upregulated in the FSBM40 group (*p* < 0.05). Among the anti-inflammatory genes, *IL4* and *TGFβ1* were remarkably downregulated in the FSBM40 group (*p* < 0.05), but not remarkably changed in the FSBM20 group (*p* > 0.05). In comparison to the FM group, *IL5* and *IL10* were both remarkably downregulated in the FSBM40 and FSBM20 groups (*p* < 0.05), and the relative expression level of *IL5* also differed remarkably between the FSBM40 and FSBM20 groups (*p* < 0.05).

**TABLE 3:** Expression of pro-inflammatory genes in the distal intestine of pearl gentian grouper fed different levels of FSBM diets (n = 3).

Gene	FM	FSBM20	FSBM40
*IL1β*	1.16 ± 0.16^a^	1.31 ± 0.03^b^	1.74 ± 0.23^c^
*IL12*	1.01 ± 0.14^a^	0.97 ± 0.18^a^	2.25 ± 0.36^b^
*IL17*	1.00 ± 0.07^a^	0.94 ± 0.12^a^	1.35 ± 0.01^b^
*TNFα*	1.01 ± 0.15^a^	1.12 ± 0.23^a^	4.09 ± 0.61^b^

Note: Data marked with the same letter indicate no significant difference.

**TABLE 4:** Expression of anti-inflammatory genes in the distal intestine of pearl gentian grouper fed different levels of FSBM diets (n = 3).

Gene	FM	FSBM20	FSBM40
*IL4*	1.01 ± 0.15^a^	0.94 ± 0.21^a^	0.48 ± 0.06^b^
*IL5*	1.00 ± 0.05^a^	0.83 ± 0.08^b^	0.40 ± 0.05^c^
*IL10*	1.02 ± 0.05^a^	0.63 ± 0.06^b^	0.56 ± 0.03^b^
*TGFβ1*	1.00 ± 0.08^a^	0.98 ± 0.09^a^	0.13 ± 0.01^b^

Note: Data marked with the same letter indicate no significant difference.

### 3.4 16S rDNA sequencing results and analysis

#### 3.4.1 Relative abundance comparison

**FIGURE 3:**
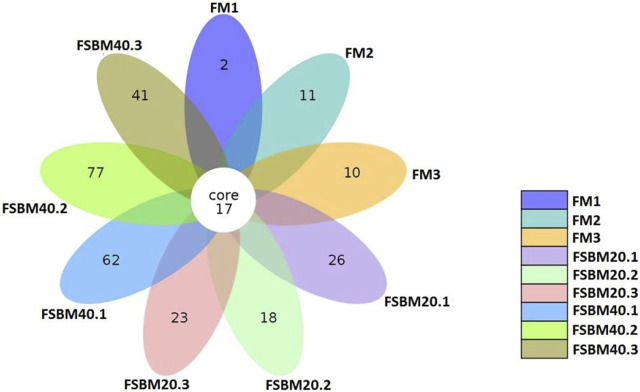
Venn diagram of OTUs of pearl gentian grouper fed different levels of fermented soybean meal diets.

**FIGURE 4:**
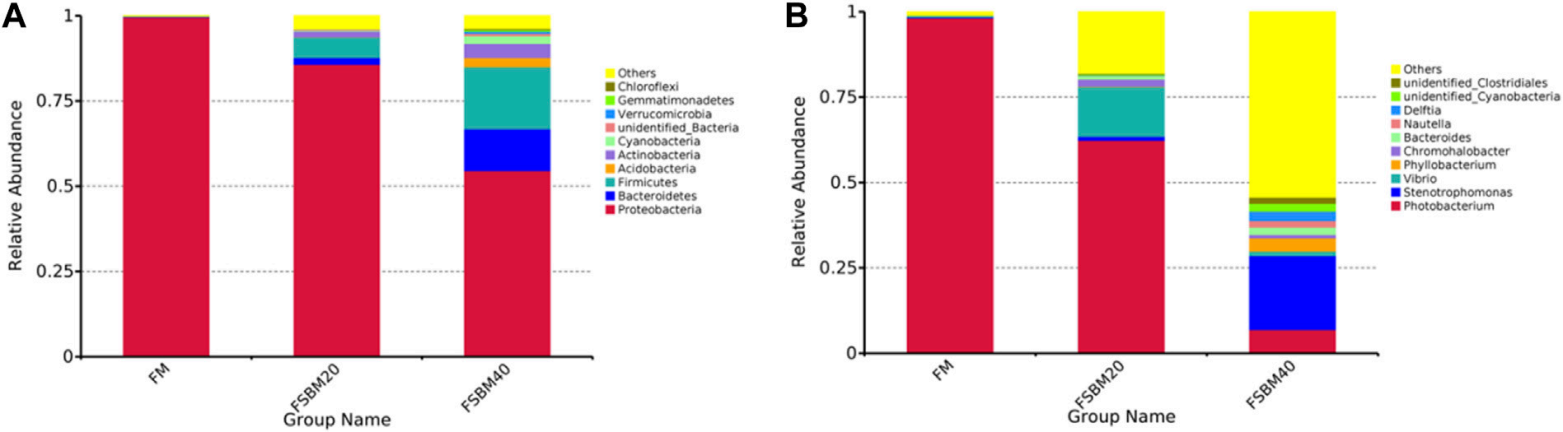
Histogram of relative abundance of gut flora OUT of pearl gentian grouper fed different levels of fermented soybean meal diets (n = 3). Note: **(A)**, phylum level;** (B)**, genus level.

After OTU clustering of the sequences with 97% similarity, 270 OTUs were detected from nine samples and 17 OTUs were shared (Figure 3). The inter-group difference between FM group and FSBM20 group was not remarkable (*p* > 0.05), but the inter-group difference with FSBM40 was remarkable (*p* < 0.05). The boxplot of species accumulation shows that the species number increased sharply as the sample number increased, and the curve flattened out (Figure 4C).

**FIGURE 5:**
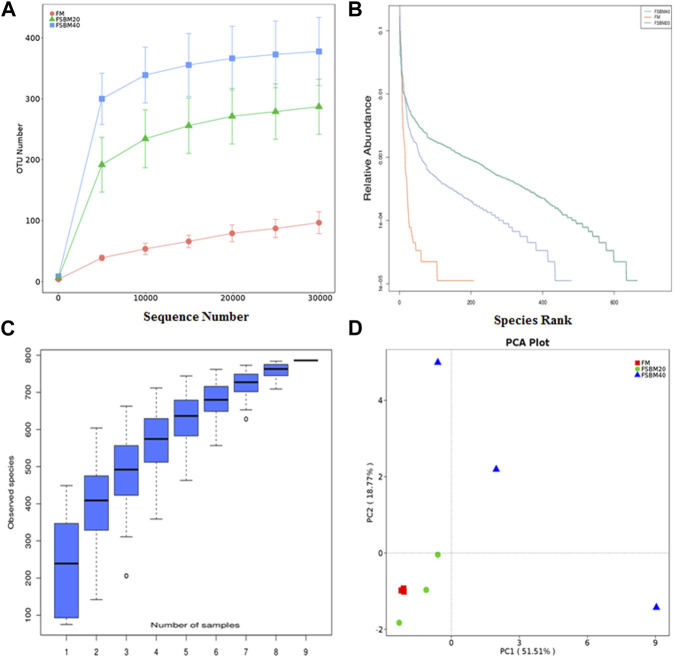
OUT diversity analysis of intestinal flora of pearl grouper fed different levels of fermented soybean meal diets. Note: **(A)**, rarefaction curve; **(B)**, rank abundance curve; **(C)**, species accumulation boxplot; **(D)**, principal component analysis.

As shown in Figure 5, *Proteobacteria* was the dominant bacteria phylum in all groups, and the proportion of *Actinobacteria*, *Firmicutes*, and *Bacteroidetes* in the FSBM20 group were remarkably higher (*p* < 0.05), and the relative abundances of *Acidobacteria*, *Actinobacteria*, *Firmicutes* and *Bacteroidetes* were further increased in the FSBM40 group (*p* < 0.05). *Photobacterium* was the dominant bacteria genus in the FM group; *Vibrio* was the dominant bacteria genus in the FSBM20 group; *Stenotrophomonas* was the dominant bacteria genus in the FSBM40 group.

#### 3.4.2 Microbial diversity analysis

**FIGURE 6:**
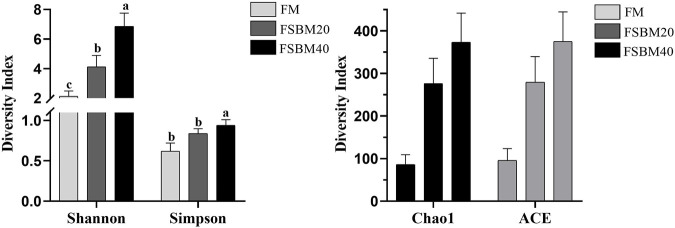
Statistical analysis of the Alpha diversity of the distal intestinal flora in pearl gentian grouper fed different levels of FSBM diets (n = 3). Note: Marked with the same letter indicates no significant difference between groups.

Alpha diversity analysis was performed on the treated OTUs and is presented as a rarefaction curve and rank abundance curves in Figures 4A, B. The alpha diversity indices increased with increasing levels of FSBM substitution (Figure 6). With respect to beta diversity, in Figure 4D, principal component analysis (PCA) indicated that the first component accounted for 51.51% of the variance and the second accounted for 18.77% of the variance. The samples from the FM group were concentrated and independent, while the samples from the FSBM20 and FSBM40 groups were dispersed, indicating that the composition of the FM group was uniform while the other groups exhibited more diverse microbial compositions.

#### 3.4.3 Associations between intestinal flora and inflammatory factors

**FIGURE 7:**
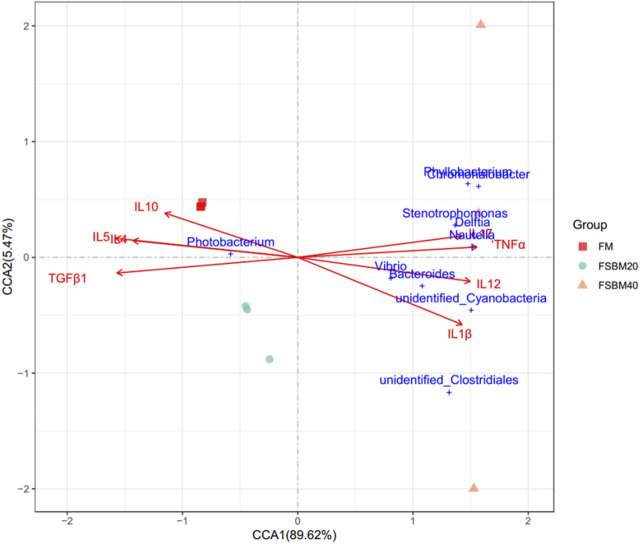
The canonical correlation analysis (CCA) between distal intestinal microbes and the inflammatory genes of pearl grouper fed different levels of fermented soybean meal diets. Note: Arrows emanating from the origin represent different inflammatory genetic factors, the length of the arrow represents the intensity of the effect of the factor on the change of the colony, and the longer the length of the arrow, the greater the effect of the factor. The angle between the arrow and the coordinate axis represents the correlation between this factor and the coordinate axis; the smaller the angle, the higher the correlation. The closer the sample point is to the arrow, the stronger the effect of this factor on the sample. Sample points located in the same direction of the arrow indicate that the factor is positively correlated with the change in the sample colony community, while samples located in the opposite direction of the arrow indicate a negative correlation.

Figure 7 shows the relationship between the inflammatory factors and Intestinal microorganisms. A positive correlation is observed when the sample points of the intestinal flora in the three groups are located in the direction of the arrow of the inflammatory factor gene; the converse is a negative correlation. The length of the arrow represents the intensity of the association between the inflammatory factors and microbial community change, with longer arrows indicating greater microbial community change. At the genus level, only *Photobacterium* was negatively correlated with *IL17*, *TNFα*, *IL12*, and *IL1β*, while the rest of the species were positively correlated with these inflammatory factors. *Photobacterium* was positively correlated with *IL10*, *IL4*, *IL5* and *TGFβ1*, while the rest of the species were negatively correlated. The envfit significance test is shown in Table 5.

**TABLE 5:** Envfit significance test for CCA association analysis of OTU abundance and inflammatory factor gene expression.

Gene	CCA1	CCA2	*r* ^2^	*P*
*IL1β*	0.9262	−0.3770	0.9007	0.002
*IL12*	0.9906	−0.1370	0.8666	0.007
*IL17*	0.9917	0.1289	0.7851	0.010
*TNFα*	0.9983	0.0575	0.9216	0.008
*IL4*	−0.9949	0.1010	0.7833	0.004
*IL5*	−0.9946	0.1036	0.9603	0.002
*IL10*	−0.9489	0.3155	0.5593	0.056
*TGFβ1*	−0.9963	−0.0859	0.9423	0.008

## 4 Discussion

In this research, the role of dietary FSBM content on the growth of aquatic animals differed from that reported in previous studies. Jeong et al. (2014) reported that high levels of FSBM in the diet could meet the growth requirements of *Oplegnathus fasciatus*. Similarly, studies of *Macrobrachium nipponense*, *Ictalurus punctatus,* and *Oncorhynchus mykiss* have suggested that it is possible to replace dietary fish meal with high levels of FSBM without adverse effects (Yamamoto et al., 2010; Ding et al., 2015). In another study of *Epinephelus coioides*, replacing less than 30% of dietary fish meal with FSBM had no significant impact on the FCR, but with greater FSBM substitution, FCR increased significantly and growth was inhibited (Shiu et al., 2015). The optimum level of FSBM dietary substitution for different species has been found to vary in studies of *Scophthalmus maximus*, *Micropterus salmoides,* and *Litopenaeus vannamei* (Wang et al., 2016; He et al., 2020; Lin and Chen, 2022). In the present study, the experimental groups fed FSBM showed remarkable differences in WGR and FCR as compared with the control group. The reasons for these differing results may include differences in feed, species and environment. The feed aspects include the fermentation strain of the FSBM, the fermentation process, and the contents of other feed ingredients. The species aspects include the feeding habits and digestive systems of the cultured species, while the environment aspects include the aquatic environment and feeding method.

Previous studies have shown that diets containing soy protein ingredients often compromise the intestinal barrier of fish and lead to intestinal dysfunction, the most common feature of which is intestinal inflammation (Krogdahl et al., 2010). The anterior intestine is considered to be the main site of nutrient absorption in teleosts, with abundant folds and microvilli in this region which increase the absorption area; the distal intestine is considered to be the key site of antigen uptake in the intestine, i.e., its immune function is more significant than the nutrient absorption process (Løkka et al., 2013). In fish nutrition experiments, subtle alterations to the digestive system tissues and cells of fish as a result of feeding with different nutrients are often observed by histopathological methods. Plant-based feeds tend to cause distal intestinal inflammation in carnivorous and omnivorous fish. Soybean meal-induced enteritis (SBMIE) in fish intestinal histology is characterised by hyperaemic mucosa, increased lamina propria thickness, shortened mucosal folds and decreased supranuclear vacuolization in epithelial cells (Sahlmann et al., 2013). In this research, similar features were observed in the distal intestinal tissues of the fish fed the FSBM diets.

SBMIE has been found in a variety of fish species including *Salmo salar*, *Danio rerio,* and pearl gentian grouper, but there are relatively few reports of fish intestinal inflammation caused by dietary FSBM, and the specific causes of such intestinal inflammation remain to be investigated (Hedrera et al., 2013; Krogdahl et al., 2015; Zhang et al., 2019). The intestinal mucosa is the first line of defence through which antigens and other substances in food need to pass, and where a variety of antioxidant and immune enzymes maintain immune homeostasis. The liver is connected to the intestine through the portal vein; gut-derived products enter the bloodstream and are mainly transported to the liver, which acts as a second line of defence to protect the intestinal mucosa from being crossed by food antigens (Brandl et al., 2017). Foodborne intestinal inflammation interferes with the immune capacity of the fish intestine and liver. Concerning gene expression, fish organism immunity is constrained by cytokine-mediated inflammatory responses (Wu et al., 2018). Changes in the gene expression levels of pro-inflammatory and anti-inflammatory cytokines can indicate inflammatory damage at the molecular level. In this study, pro-inflammatory cytokines, including *IL17*, and anti-inflammatory cytokines, such as *IL10*, were significantly upregulated and downregulated, respectively, in the intestines of the FSBM40 group; this is similar to many studies of intestinal inflammation in various fish species (Marjara et al., 2012; Torrecillas et al., 2015). The intestine of pearl gentian grouper responds to dietary FSBM and that the inflammation triggered by this diet may be lymphocyte-mediated and lymphokine-driven.

Plant-based ingredients are detrimental to the intestinal absorption of carnivorous fish, and may induce intestinal inflammation (Liu et al., 2018; Willora et al., 2022; Zhang et al., 2023). In addition to the effect of ANFs, it is suspected that a variety of other factors combine to influence the maintenance of normal intestinal physiology. Studies have shown that the intestinal microbes and the various metabolites produced by the metabolism of different microorganisms are tightly connected with intestinal inflammation in animals (Nayak, 2010). *Bacillus subtilis* has been used as a probiotic strain in aquaculture. In this study, FSBM derived from *Bacillus subtilis* fermentation was used in the experimental group diets to explore the role of FSBM on the intestinal flora of the fish. *Bacillus subtilis* was added to the SBM diet of fish and it was found that *Bacillus subtilis* benefited the growth of fish and improved its antioxidant capacity (Li et al., 2019). In studies of *Litopenaeus vannamei* and *Oreochromis niloticus*, feeding *Bacillus subtilis*-containing diets was found to be beneficial to the health of the fish (Li et al., 2009; Hassaan et al., 2014). Zhou et al. (2019) stimulated *Ctenopharyngodon idella* dendritic cells (DCs) with UV-irradiated inactivated probiotic *Bacillus subtilis* under *in vitro* conditions, and the gene expression levels of *IL4* and *IL10* were increased. This finding suggested that *Bacillus subtilis* can affect the immune function of *Ctenopharyngodon idella* organisms by promoting the secretion of anti-inflammatory cytokines from DCs.

In this research, 16S rDNA sequencing was used to examine the role of feeding FSBM on the intestinal flora of pearl gentian grouper. This indicated that the intestinal microbiota composition changed after exposure to the FSBM diet, leading to an increase in diversity and abundance. This may be due to pathogen infection or antigen exposure which, in turn, affects intestinal microbes (Sanmukh et al., 2012). An increase in the diversity and abundance of potentially pathogenic bacteria produces intestinal dysfunction. The composition of the microbiota of the fish intestine is remarkably different from that of mammals. In general, the intestinal microbial population of fish consists of two major phyla, *Proteobacteria* and *Firmicutes* (Navarrete et al., 2010). This is the same as the results of the intestinal flora analysis obtained in this study. He et al. (2020) fed *Micropterus salmoides* a diet containing FSBM instead of 30% fish meal and found that the abundance of *Proteobacteria* in the fish intestine was remarkably changed. Similarly, Miao et al. (2018) studied *Channa argus* and found that feeding soybean meal resulted in a significant decrease in the proportion of *Proteobacteria* in the intestine. At the genus level, *Aeromonas*, *Alcaligenes*, *Alteromonas*, *Carnobacterium*, *Flavobacterium*, *Micrococcus*, *Moraxella*, *Photobacterium*, *Pseudomonas* and *Vibrio* are the dominant intestinal microorganisms in various marine fish species (Pérez et al., 2010). In this study, *Photobacterium* was found to be the dominant intestinal bacterium in the FM group, and *Photobacterium* was remarkably decreased in the FSBM40 group. In the FSBM40 group, *Stenotrophomonas* remarkably increased and became the most dominant bacterium. The *Photobacterium* genus, which belongs to the family Vibrionaceae, is a common microbial species discovered in marine environments, on the surface of marine animals, and in the digestive tracts of marine animals (Labella et al., 2018). This species can live in both anaerobic and aerobic environments and is present as a symbiont in some fish luminescent organs. ANFs not completely eliminated in FSBM and beneficial microorganisms in FSBM may underlie the inhibition of *Photobacterium* proliferation and the increase in intestinal flora diversity.

The immune function of biological organisms and the microbial community are regulated by each other, and immunity influences the establishment of the intestinal flora. For example, T lymphocytes regulate the abundance of *Vibrio* in the intestine (Brugman et al., 2015). It is possible that alterations in microbial communities could be a cause of intestinal inflammation. The association analysis between the gene expression levels of inflammatory cytokines and the genus-level abundance of intestinal flora showed associations between the abundance of several relatively dominant bacteria at the genus level and inflammatory cytokines. Microorganisms of *Photobacterium* gradually decreased in the FM, FSBM20 and FSBM40 groups, while microorganisms of *Stenotrophomonas* and *Vibrio* were remarkably reduced in the FM group compared to FSBM20 and FSBM40 groups. This corresponds to the decreased expression levels of anti-inflammatory genes and the increased expression levels of pro-inflammatory genes in the FSBM-replacement groups. Such results indicate that changes in the gut microbiota caused by FSBM were accompanied by the synthesis and secretion of anti-inflammatory cytokines, including *IL10*, and pro-inflammatory cytokines, such as *IL17*. This may be the result of a combination of causes. For example, short-chain fatty acids could further influence the development of intestinal inflammation by regulating regulatory T cells (Smith et al., 2013). Indole, a substance produced by the metabolism of tryptophan by intestinal microorganisms, contributed to the alleviation of intestinal inflammation in the gut, as evidenced by regulation of proinflammatory genes, increased expression of anti-inflammatory genes, enhanced epithelial barrier properties, and reduced pathogen colonization (Bansal et al., 2010). The role of these bacterial metabolites is not negligible. The exact manner of association between other intestinal flora and intestinal inflammation remains to be investigated.

## 5 Conclusion

In summary, this research demonstrates that the massive substitution of fish meal with FSBM is not conducive to the growth of pearl gentian fish and produces changes in their intestinal morphology that are not conducive to proper function. In addition, FSBM disturbed the gut microbial balance of the fish and triggered abnormal expression of inflammatory cytokines. These findings can inform future research on the mechanisms and influencing factors of food-borne intestinal inflammation in this species, which will be beneficial to the conservation of fish meal resources and the promotion of green and sustainable aquaculture.

## Data Availability

The data presented in the study are deposited in the NCBI Sequential Read Archive (SRA) repository, accession number PRJNA666309.
