# Supplementary figures and images for: Effects of fermented soybean meal substitution for fish meal on intestinal flora and intestinal health in pearl gentian grouper
Source: Front Physiol. 2023 Jul 3;14:1194071. doi: 10.3389/fphys.2023.1194071 (PMC10352108; doi:10.3389/fphys.2023.1194071)

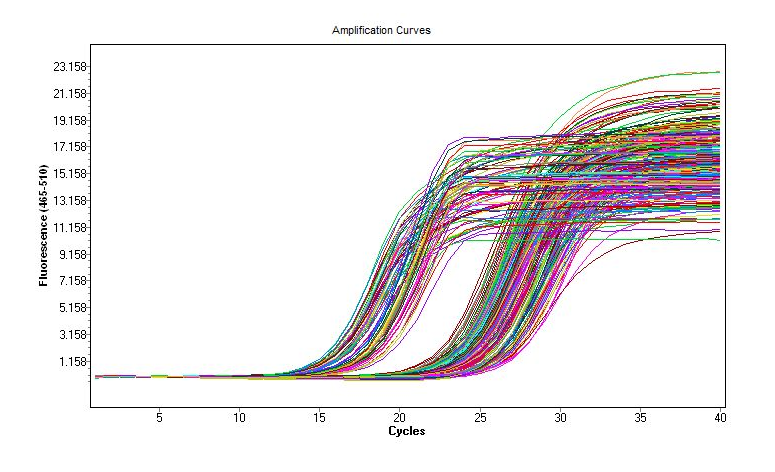


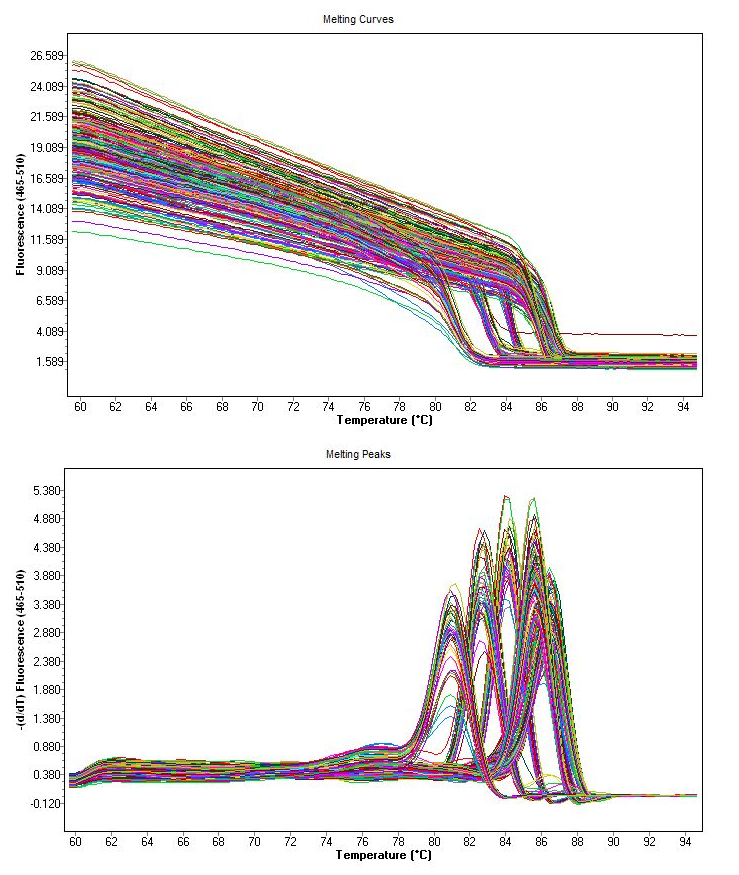

Supplement: Supplementary file 1 [file DataSheet1.docx]
